# Prediction of ^177^Lu-DOTATATE Therapy Outcomes in Neuroendocrine Tumor Patients Using Semi-Automatic Tumor Delineation on ^68^Ga-DOTATATE PET/CT

**DOI:** 10.3390/cancers16010200

**Published:** 2023-12-31

**Authors:** Hwan Lee, Sarit T. Kipnis, Remy Niman, Sophia R. O’Brien, Jennifer R. Eads, Bryson W. Katona, Daniel A. Pryma

**Affiliations:** 1 Department of Radiology, University of Pennsylvania Perelman School of Medicine, Philadelphia, PA 19104, USA; 2 Department of Medicine, University of Pennsylvania Perelman School of Medicine, Philadelphia, PA 19104, USA; 3 Department of Medicine, Georgetown University, Washington, DC 20007, USA; 4 MIM Software Inc., Cleveland, OH 44122, USA; 5 Abramson Cancer Center, University of Pennsylvania, Philadelphia, PA 19104, USA

**Keywords:** neuroendocrine tumor, ^68^Ga-DOTATATE, positron emission tomography computed tomography, ^177^Lu-DOTATATE, prognostic factor

## Abstract

**Simple Summary:**

^177^Lu-DOTATATE is a radioactive drug that can treat advanced neuroendocrine tumors, a type of cancer. However, some patients do not benefit from ^177^Lu-DOTATATE treatment and there is an unmet need to identify such patients before they receive the treatment. Currently, each patient needs to have a positive result on a scan, such as ^68^Ga-DOTATATE PET/CT, prior to receiving ^177^Lu-DOTATATE treatment to verify that the drug can attack the patient’s cancer. This study used a semi-automatic analysis of ^68^Ga-DOTATATE PET/CT to predict the results of ^177^Lu-DOTATATE treatment. Having a large amount of cancer and/or a tumor with low signal on ^68^Ga-DOTATATE PET/CT predicted poor results of ^177^Lu-DOTATATE therapy. The semi-automatic nature of this method allows identification of patients at risk for treatment failure with little time and effort.

**Abstract:**

Background: Treatment of metastatic neuroendocrine tumors (NET) with ^177^Lu-DOTATATE peptide receptor radionuclide therapy (PRRT) results in favorable response only in a subset of patients. We investigated the prognostic value of quantitative pre-treatment semi-automatic ^68^Ga-DOTATATE PET/CT analysis in NET patients treated with PRRT. Methods: The medical records of 94 NET patients who received at least one cycle of PRRT at a single institution were retrospectively reviewed. On each pre-treatment ^68^Ga-DOTATATE PET/CT, the total tumor volume (TTV), maximum tumor standardized uptake value for the patient (SUVmax), and average uptake in the lesion with the lowest radiotracer uptake (SUVmin) were determined with a semi-automatic tumor delineation method. Progression-free survival (PFS) and overall survival (OS) among the patients were compared based on optimal cutoff values for the imaging parameters. Results: On Kaplan–Meier analysis and univariate Cox regression, significantly shorter PFS was observed in patients with lower SUVmax, lower SUVmin, and higher TTV. On multivariate Cox regression, lower SUVmin and higher TTV remained predictive of shorter PFS. Only higher TTV was found to be predictive of shorter OS on Kaplan–Meier and Cox regression analyses. In a post hoc Kaplan–Meier analysis, patients with at least one high-risk feature (low SUVmin or high TTV) showed shorter PFS and OS, which may be the most convenient parameter to measure in clinical practice. Conclusions: The tumor volume and lowest lesion uptake on ^68^Ga-DOTATATE PET/CT can predict disease progression following PRRT in NET patients, with the former also predictive of overall survival. NET patients at risk for poor outcomes following PRRT can be identified with semi-automated quantitative analysis of ^68^Ga-DOTATATE PET/CT.

## 1. Introduction

Neuroendocrine tumors (NETs) represent a heterogeneous group of neoplasms with rising incidence and prevalence [1]. NETs are typically gastroenteropancreatic or pulmonary in origin, and the remaining 5–13% arise from unknown or various rare locations throughout the body [2,3,4]. Approximately 30% of NET patients present with metastatic disease, putting emphasis on the role of systemic therapy [2]. The most widely used molecular target for NETs is the somatostatin receptor (SSTR), particularly SSTR subtype 2 which is overexpressed in approximately 80% of NETs [5]. Radiolabeled small molecules targeting SSTR have been successfully developed for both diagnostic and therapeutic applications in NETs.

One such therapeutic application is peptide receptor radionuclide therapy (PRRT) with beta particle-emitting ^177^Lu-DOTATATE. Since its clinical approval by the United States Food and Drug Administration in 2018, it has been recognized as a key systemic treatment option for SSTR-positive NETs [6]. Its approval was based on the landmark NETTER-1 trial, where PRRT conferred PFS benefit in grade 1–2 midgut NET patients with a hazard ratio of 0.21 compared to a randomized control group treated with long-acting octreotide [7]. However, heterogeneous response to PRRT has been observed in the NET patient population [8]. The NETTER-1 trial showed a modest objective response rate of 18%, and subsequent prospective studies demonstrated lack of PFS benefit in a subset of NET patients [7,9,10]. Therefore, there is an unmet need to prospectively identify which NET patients will derive the most benefit (or are unlikely to benefit) from PRRT.

There has been active research in identifying clinical, imaging, laboratory, and pathologic predictors of post-PRRT outcomes. For example, Das et al. previously reported the clinical score, which ranges from 0–10 based on clinical symptoms, tumor distribution, primary tumor site, and prior therapy. The authors found that NET patients with higher Clinical Score were more likely to demonstrate shorter PFS and OS following PRRT [11]. Bodei et al. developed the PRRT Prediction Quotient, a peripheral blood-based genomic assessment that could predict response to PRRT with 96% accuracy [12]. A recent meta-analysis by Lee et al. identified 15 studies that investigated the value of pre-treatment PET/CT for post-PRRT prognosis [13].

Prior to initiation of PRRT, functional imaging of NETs with SSTR-targeted positron emission tomography (SSTR-PET) using ^68^Ga- or ^64^Cu-labeled DOTATATE, DOTATOC, or DOTANOC is routinely performed for confirmation of tumor SSTR positivity [14]. The ability of SSTR-PET to show quantitative tumor SSTR expression and its availability in virtually every patient receiving PRRT support its use as a predictor of post-PRRT outcomes [15]. Early studies on this topic have focused on the maximum standardized uptake value (SUVmax) on SSTR-PET, in which higher patient-level SUVmax was predictive of favorable post-PRRT outcomes [16,17]. More recently, a study on NET patients not limited to those who received PRRT showed that the value of SSTR-PET for general prognosis was greatly increased by using the average SUV of the least avid lesion (SUVmin) rather than patient-level SUVmax [18]. The study used a combination of SUVmin and total tumor volume (TTV) to best predict PFS and OS [18].

In the present study, we examined the prognostic value of SUVmin and TTV, in addition to SUVmax, specifically in NET patients undergoing PRRT. The time requirement and variability in manually contouring the entire tumor burden for each patient make it difficult, if not prohibitive, to translate volumetric PET parameters to routine clinical practice. The aforementioned study as well as several recent studies on SSTR-PET used a semi-automatic tumor delineation method to overcome this challenge [18,19,20]. We applied a semi-automatic tumor contouring method on ^68^Ga-DOTATATE PET/CT in order to develop a clinically feasible method of patient stratification for PRRT, with emphasis on identifying a subset of NET patients who are less likely to benefit from PRRT.

## 2. Materials and Methods

### 2.1. Study Population

Approval from the University of Pennsylvania Institutional Review Board was obtained prior to the study, and the requirement for informed consent was waived. The pre-treatment ^68^Ga-DOTATATE PET/CT and medical records of 128 consecutive NET patients who received at least one cycle of ^177^Lu-DOTATATE treatment at the University of Pennsylvania between August 2018 and February 2022 were reviewed retrospectively. In order to minimize variability in image analysis, 28 patients who obtained their pre-treatment ^68^Ga-DOTATATE PET/CT outside the University of Pennsylvania were excluded from the study. An additional six patients were excluded due to the lack of available follow-up imaging. A total of 94 patients were included in the study.

### 2.2. Patient Assessments

The baseline characteristics of the study population were collected, including gender, age, primary tumor site, tumor grade, and Ki-67 index. Individual tumors on the pre-treatment ^68^Ga-DOTATATE PET/CT for each patient were contoured semi-automatically as previously described [21]. Briefly, a 3 cm diameter spherical volume of interest was placed in a part of the liver without any tumor, and individual lesions were contoured automatically based on the SUV threshold of 1.5 times the mean plus two standard deviations. The volume of interest and SUV threshold were based on the PET Response Criteria in Solid Tumors [22], which were successfully adopted by other studies that used semi-automatic tumor delineation [18,20,21]. After manually removing areas of physiologic uptake, SUVmax, SUVmin, and TTV were extracted from the resulting tumor contours. A minimum tumor volume threshold of 2 mL was applied to minimize partial volume effect. MIM version 7 (MIM Software Inc., Cleveland, OH, USA) was used for image analysis. OS and RECIST 1.1-based PFS for each patient were calculated relative to the first cycle of PRRT [23].

### 2.3. Statistical Analysis

The baseline characteristics of the study population were summarized with descriptive statistics. The best cutoff values for SUVmax, SUVmin, and TTV were determined with respect to PFS based on the minimum *p*-value approach [24]. PFS and OS with respect to the SUVmax, SUVmin, and TTV cutoffs were examined with Kaplan–Meier survival analysis, where Mantel-Cox test was used for identification of statistically significant differences in survival. In addition, the prognostic value of the SUVmax, SUVmin, and TTV cutoffs for PFS and OS was examined using univariate and multivariate Cox regression with 5% entry probability and 10% removal probability.

All statistical tests were two-sided and *p*-values less than 0.05 were considered statistically significant. The statistical analyses were performed using GraphPad Prism 8 (GraphPad Software, San Diego, CA, USA), except for Cox regression which was performed with IBM SPSS Statistics 25 (SPSS Inc., Chicago, IL, USA).

## 3. Results

### 3.1. Patient Characteristics

The baseline characteristics of the study population are summarized in Table 1. Out of 94 patients, 47 patients (50%) were female, with a median age of 61 years (range: 19–89). Small intestinal (36%) and pancreatic (35%) NETs were most common. The median Ki-67 index was 8%, ranging from 0% to 95%. Approximately three-quarters of patients received all four cycles of PRRT (Table 1).

### 3.2. Imaging Parameters

**Figure 1:**
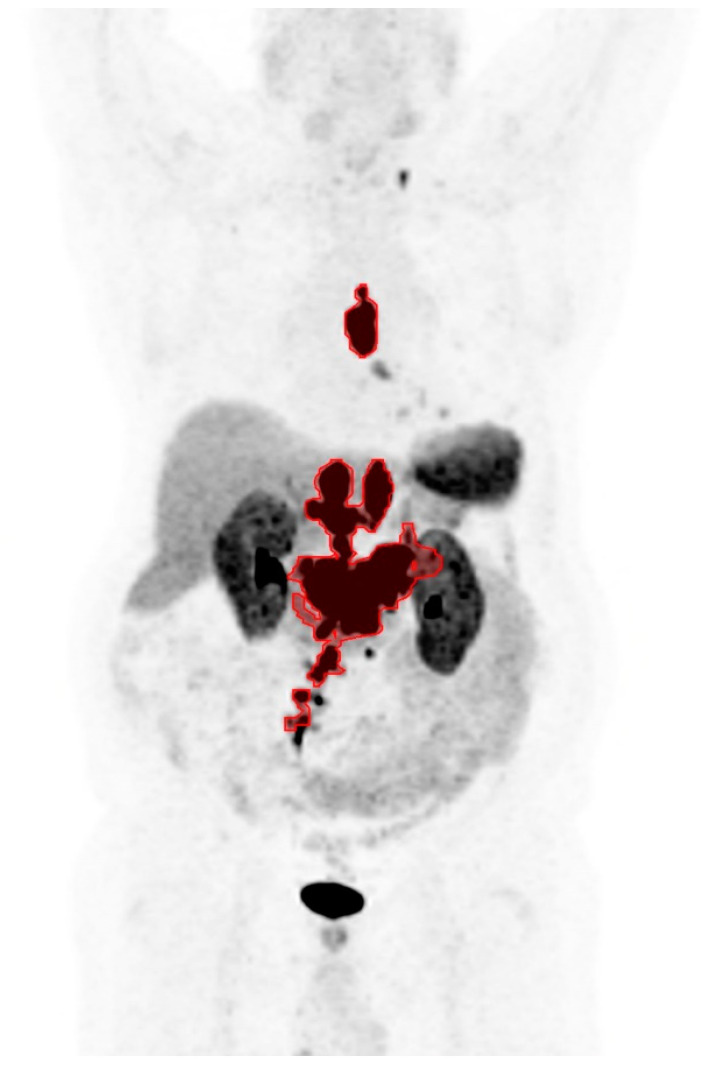
A coronal maximum intensity projection image of ^68^Ga-DOTATATE PET showing an example of the semi-automatic tumor delineation in red.

An example of the semi-automatic tumor delineation is shown as a coronal maximum intensity projection image in Figure 1: the tumor lesions are marked in red, areas of physiologic uptake are excluded, and tumor lesions below the size or uptake threshold are also excluded. Although all patients in the study had SSTR-positive tumors on ^68^Ga-DOTATATE PET/CT which made them eligible for PRRT, no lesion was detected in six patients due to subthreshold level tumor radiotracer uptake. The six patients were therefore assigned SUVmax and SUVmin of zero for stratification into the low SUV groups. The optimal cutoff values for SUVmax, SUVmin, and TTV were 20.4 (median: 45.0), 6.7 (median: 9.9), and 325 mL (median: 160 mL), respectively.

### 3.3. Follow-Up

**Figure 2:**
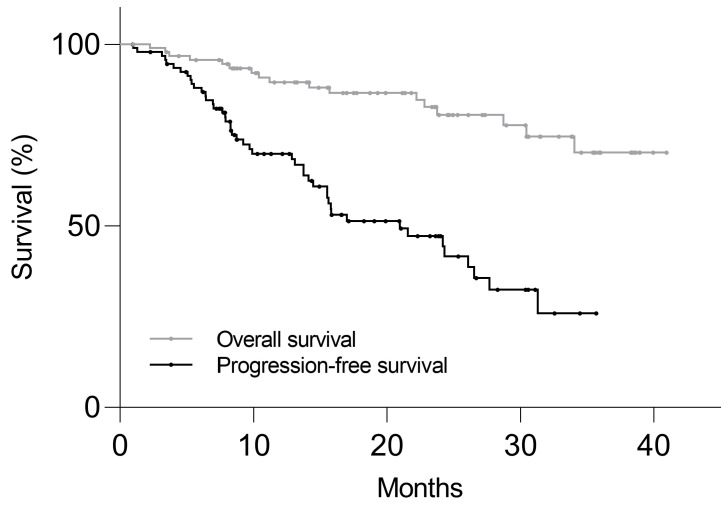
Kaplan–Meier plot of progression-free survival and overall survival in 94 NET patients who received PRRT. Median progression-free survival was 21.0 months and median overall survival was not reached.

From the first cycle of PRRT, the median duration of clinical follow-up was 21.3 months (range: 1.0–41.0 months). Figure 2 shows the Kaplan–Meier plot of PFS and OS in the study population. There were 46 (49%) patients with disease progression, with the median PFS of 21.0 months. Progression-free probability at 6, 12, and 24 months was 88 ± 3%, 70 ± 5%, and 47 ± 6%, respectively (mean ± SEM). There were 17 deceased patients (18%) by the last follow-up, and the median OS was not reached.

### 3.4. Survival Analysis

**Figure 3:**
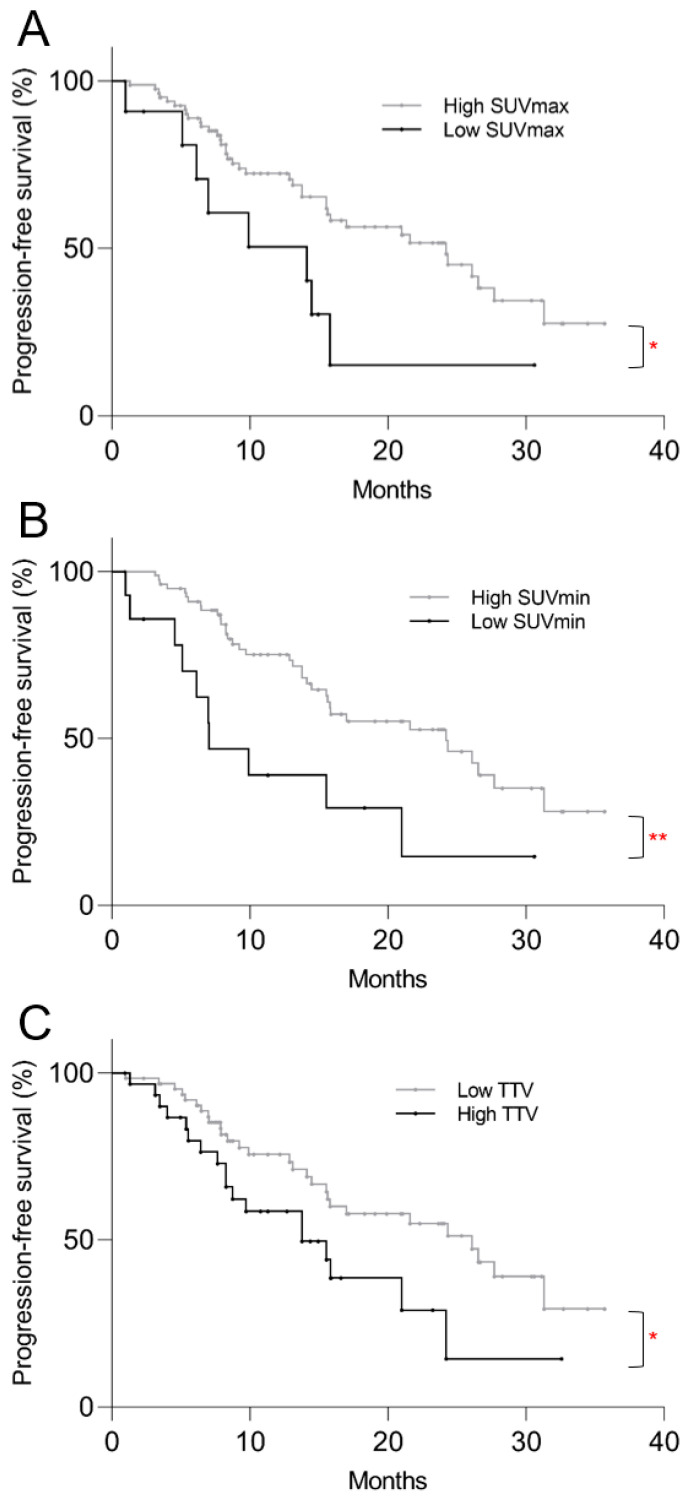
Kaplan–Meier plots of post-PRRT progression-free survival based on pre-treatment ^68^Ga-DOTATATE PET/CT parameters. Progression-free survival was significantly shorter in the (**A**) low SUVmax (*p* = 0.032), (**B**) low SUVmin (*p* = 0.006), and (**C**) high TTV (*p* = 0.034) groups. * *p* < 0.05, ** *p* < 0.01.

The Kaplan–Meier survival analysis revealed significantly shorter PFS in the low SUVmax group compared to the high SUVmax group, with median of 14.1 vs. 24.2 months, respectively (*p* = 0.032, Figure 3A). Significantly shorter PFS was also found in the low SUVmin group (7.0 vs. 24.2 mo, *p* = 0.006) and high TTV group (13.8 vs. 26.1 mo, *p* = 0.034), as shown on Figure 3B,C. On univariate Cox regression analysis, low SUVmax (*p* = 0.037), low SUVmin (*p* = 0.008), and high TTV (*p* = 0.037) were predictive of shorter PFS (Table 2). On multivariate analysis, low SUVmin (*p* = 0.030) and high TTV (*p* = 0.007) remained predictive of shorter PFS.

**Figure 4:**
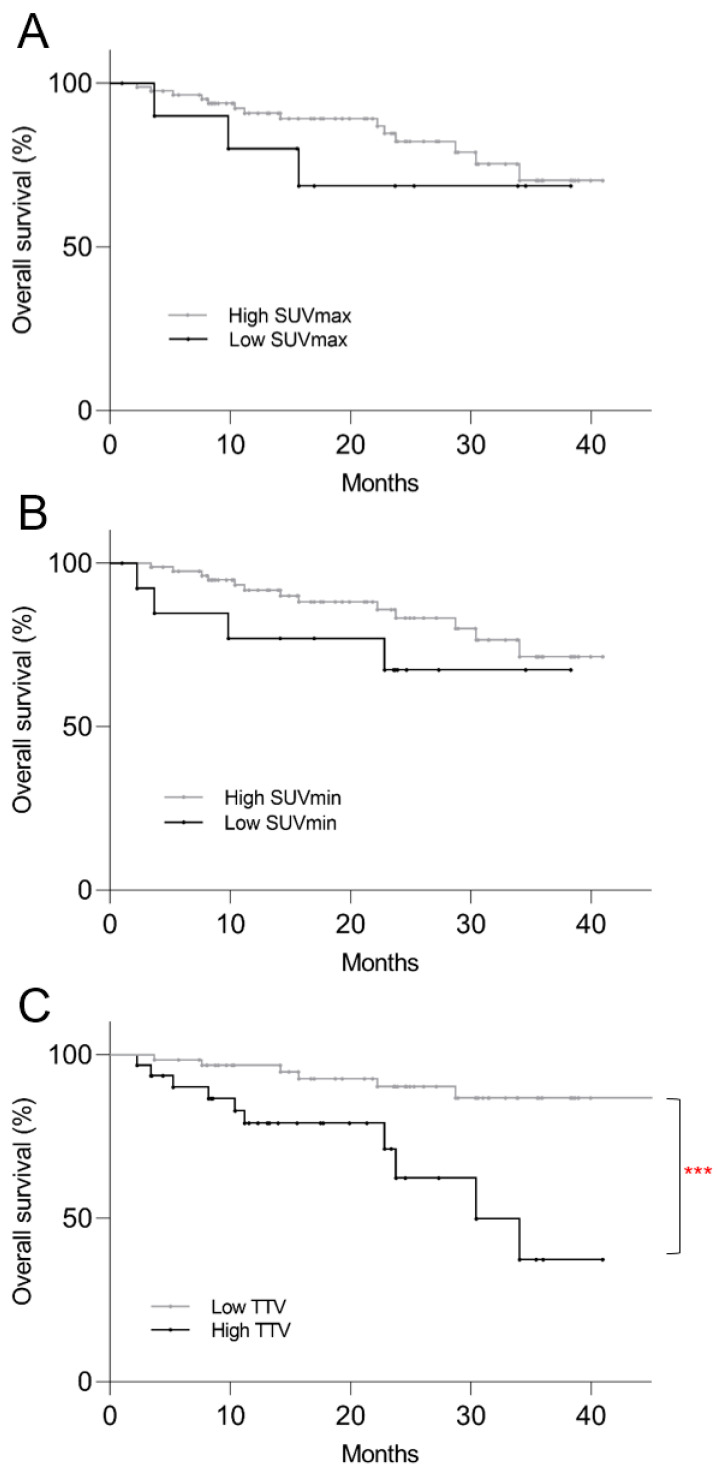
Kaplan–Meier plots of post-PRRT overall survival based on pre-treatment ^68^Ga-DOTATATE PET/CT parameters. (**A**) Low SUVmax (*p* = 0.40) and (**B**) low SUVmin (*p* = 0.22) were not predictive of mortality risk, but patients with (**C**) higher TTV showed significantly shorter overall survival (*p* < 0.001). *** *p* < 0.001.

On the Kaplan–Meier survival analysis for OS, low SUVmax (*p* = 0.40) and low SUVmin (*p* = 0.22) were not predictive of mortality risk, but patients with higher TTV showed significantly shorter OS (*p* < 0.001) (Figure 4). Only higher TTV was predictive of mortality risk on univariate regression analysis (*p* = 0.006) and multivariate regression analysis (*p* = 0.003) (Table 3).

### 3.5. Post Hoc Kaplan–Meier Analysis

**Figure 5:**
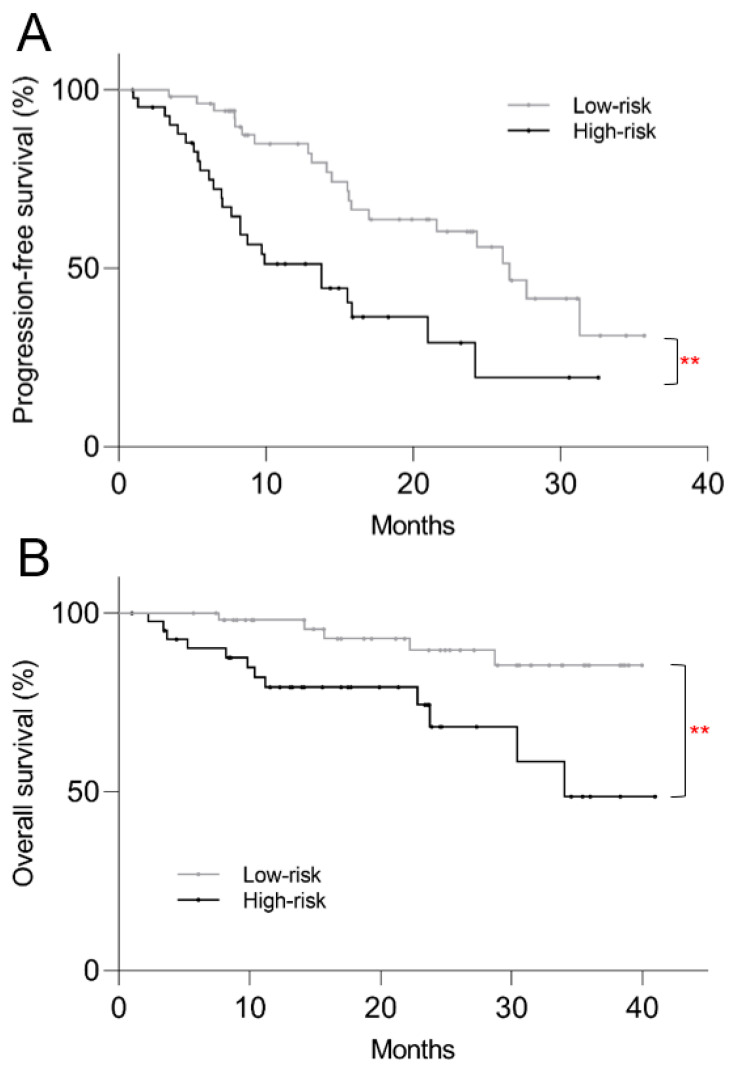
Kaplan–Meier plots of post-PRRT survival based on high vs. low-risk stratification using pre-treatment ^68^Ga-DOTATATE PET/CT. High-risk patients showed significantly shorter (**A**) progression-free survival (*p* = 0.001) and (**B**) overall survival (*p* = 0.006). ** *p* < 0.01.

In order to provide a single method of patient stratification for translation into routine clinical practice, patients were divided into high-risk and low-risk groups. The high-risk group was defined by presence of at least one of the two risk factors for progression: low SUVmin and/or high TTV. On a post hoc Kaplan–Meier survival analysis, the high-risk group showed shorter PFS (median 13.8 mo vs. 26.6 mo, *p* = 0.001) and OS (34.1 mo vs. >40 mo, *p* = 0.006) compared to the low-risk group (Figure 5).

## 4. Discussion

The present study examined the prognostic value of pre-treatment ^68^Ga-DOTATATE PET/CT in NET patients undergoing PRRT, specifically examining lesion avidity and tumor volume. SUVmin and tumor volume were found to be superior to SUVmax in predicting post-PRRT outcomes. Emphasis was placed on translation of the results into clinical practice by using a semi-automated tumor contouring method which takes only a few minutes per scan and by integrating the results into a single stratification method for ease of application.

The improved prognostic value of using SUVmin and TTV over SUVmax in the post-PRRT setting is similar to what was found in the previous study by Carlsen et al. for general prognosis in NET patients [18]. Studies using lesion-level analysis found that individual tumors with higher radiotracer uptake on SSTR-PET showed favorable outcomes following PRRT, suggesting that disease progression following PRRT is driven by the lesions with low SUV in a given patient [25,26]. From a tumor biology perspective, the least avid lesion in a given patient not only has the lowest target receptor density for PRRT [15], but also is more likely to have a more aggressive phenotype relative to other tumor lesions with higher SSTR expression [27]. Therefore, our approach using the least avid lesion uptake for prognosis is in keeping with the current understanding of NET biology. Although SUVmax is easy to measure and provides prognostic information, our study specifically supports the use of SUVmin and TTV given the improved insights into the patient’s overall disease phenotype offered by the volumetric PET parameters.

The specific SUVmin cutoff value of 6.7 we found is lower than the value of 14.2 used in the study by Carlsen et al. and can be attributed to differences in the PET radiotracer, cutoff determination method, evaluated outcomes, and patient characteristics [18]. For TTV, previous studies on post-PRRT outcomes reported variable cutoffs ranging from 141 mL to 672 mL [19,28,29,30], and our cutoff of 325 mL falls within this range. Although SUVmax was not a significant predictor of PFS on multivariate regression due to co-variation with SUVmin in our study, it still contains prognostic information when used alone. The SUVmax cutoff of 20.4 we found is slightly higher than previously reported values of 13.0–17.9 [16,17,25,31]. The variation in the reported cutoffs illustrate the heterogeneity in both the patient cohorts and SUV measurements across studies [13,32]. While we report the general principle of identifying high-risk patients using SUVmin and TTV, institution-specific cutoffs should be determined for generalizability beyond our study.

Beyond the parameters evaluated in our study, there is an evolving body of research on volumetric analysis of SSTR-PET for prediction of post-PRRT outcomes. Radiomic analysis of the entire tumor volume for extraction of textural features, for example tumor heterogeneity, has been successful in predicting post-PRRT outcomes in several studies [33,34,35]. Decrease in tumor SUV after initiation of PRRT has been also associated with favorable outcomes [36]. While analysis of radiomic features or post-treatment PET is beyond the scope of our study that focused on developing a simple pre-treatment stratification method, the prior studies add to the insight that SSTR-PET can offer more information than confirmation of tumor SSTR positivity for PRRT eligibility. It is worth noting that there is also a considerable body of literature reporting the lack of prognostic value of the same SSTR-PET parameters [37,38,39,40]. Given that most studies that used SSTR-PET for prognostication of post-PRRT outcomes were retrospectively conducted with small sample sizes [13], larger prospective studies are needed for validation of the predictive SSTR-PET parameters for PRRT.

The present study is also subject to the limitations of a single-center retrospective design. Partial volume effect was reduced but not eliminated by our application of the minimum tumor size threshold, where a compromise between lesion detection and SUV measurement had to be made [18,41]. The semi-automatic tumor delineation method had drawbacks compared to manual contouring. First, it combined contiguous lesions into a single lesion, which would not affect TTV but would potentially increase SUVmin if a patient’s least avid lesion is combined with an adjacent more avid lesion. Second, it missed tumor lesions with low-level radiotracer uptake, which would lead to underestimation of TTV and overestimation of SUVmin. Despite the limitations, the semi-automatic tumor delineation method yielded meaningful prognostic information in our patient cohort and the benefits of using semi-automated tumor delineation on efficiency and standardization are well acknowledged in the literature. For example, previous studies showed that inter-observer variability in tumor contouring is a potential barrier to radiomic analysis of oncologic PET, and semi-automatic tumor delineation reduced the variability [42,43]. Each PET exam can be analyzed in as quickly as 5 min with semi-automatic tumor contouring [18], consistent with our experience in the present study. Finally, since our study relied on semi-automatic analysis of a single pre-treatment ^68^Ga-DOTATATE PET/CT for each patient, it did not incorporate other imaging studies such as CT, MRI, or ^18^F-fluorodeoxyglucose PET. Additional tumor lesions identified on other imaging modalities would potentially increase TTV and decrease SUVmin, at the expense of manual cross-referencing and tumor contouring.

The limitations of semi-automatic tumor delineation may be overcome by artificial intelligence algorithms that achieve accurate and reproducible tumor contouring with complete automation [44]. Artificial intelligence also has the potential to build a single prediction model based on various parameters such as clinical data, pathology results, multi-modality imaging, and radiomic features [45]. The prognostic value of semi-automatic PET analysis we found in the present study supports the development of artificial intelligence algorithms as the ultimate goal for more advanced prediction of post-PRRT outcomes.

## 5. Conclusions

We successfully developed a clinically feasible method of patient stratification for predicting post-PRRT outcomes using a semi-automatic tumor delineation method on SSTR-PET. We found that the tumor volume and least avid lesion uptake on ^68^Ga-DOTATATE PET/CT can predict early disease progression following PRRT in NET patients, with the former also predictive of mortality.

In order to make a fully informed choice of PRRT, a comparison to a control group of patients is needed to estimate the expected benefit of PRRT [7,9]. Despite the absence of a control group, our study provides clinical utility in identification of patients who may have poor outcomes following PRRT. Since PRRT is deemed effective only until subsequent disease progression, the median PFS we observed in the high-risk group represents the maximum possible median PFS benefit gained from PRRT in this group compared to a hypothetical control group without PRRT. This knowledge of expected earlier disease progression in the high-risk group can foster personalized management decisions. For instance, earlier anatomic assessment of disease progression may be pursued or perhaps treatment with a different systemic therapy may be chosen over PRRT.

In the future, our prognostic approach would benefit from further validation in a prospective, multi-center setting. Ultimately, artificial intelligence-based predictive modeling of post-PRRT outcomes in combination with other independent clinical, imaging, laboratory, and pathologic predictors may help clinicians and patients make a more informed choice when considering PRRT.

## Figures and Tables

**Table 1:** Baseline characteristics of the study population.

Baseline Characteristics	Percentage (n = 94)
Gender	
Male	50%
Female	50%
Age (years)	
≤50	17%
>50 and ≤60	31%
>60 and ≤70	26%
>70	27%
Primary tumor site	
Small intestine	36%
Pancreas	35%
Colon	9%
Lung	9%
Other gastrointestinal	3%
Pheochromocytoma/Paraganglioma	2%
Unknown	6%
Grade for gastroenteropancreatic tumors	
G1	28%
G2	61%
G3	11%
Ki-67 index	
Median	8%
Range	0–95%
Number of ^177^Lu-DOTATATE cycles	
4	74%
3	11%
2	7%
1	7%

**Table 2:** Predictors of post-PRRT progression on pre-treatment ^68^Ga-DOTATATE PET/CT.

Parameter	Univariate			Multivariate		
	HR	95% CI	*p*-Value	HR	95% CI	*p*-Value
Low SUVmax	2.3	1.0–4.9	**0.037**	1.5	0.6–4.0	0.418
Low SUVmin	2.8	1.3–6.1	**0.008**	3.1	1.1–8.7	**0.030**
High TTV	1.9	1.0–3.5	**0.037**	2.4	1.3–4.7	**0.007**

HR = hazard ratio; CI = confidence interval. Values in bold indicate *p* < 0.05.

**Table 3:** Predictors of post-PRRT mortality on pre-treatment ^68^Ga-DOTATATE PET/CT.

Parameter	Univariate			Multivariate		
	HR	95% CI	*p*-Value	HR	95% CI	*p*-Value
Low SUVmax	1.7	0.5–5.9	0.412	1.7	0.3–9.1	0.530
Low SUVmin	1.7	0.5–6.0	0.393	3.0	0.5–18.1	0.233
High TTV	3.9	1.5–10.3	**0.006**	5.9	1.8–18.7	**0.003**

HR = hazard ratio; CI = confidence interval. Values in bold indicate *p* < 0.05.

## Data Availability

The data are not publicly available for protection of patient privacy under the terms of the study’s ethical approval.
